# Broad Cross-Reactive Epitopes of the H5N1 Influenza Virus Identified by Murine Antibodies against the A/Vietnam/1194/2004 Hemagglutinin

**DOI:** 10.1371/journal.pone.0099201

**Published:** 2014-06-19

**Authors:** Mie Kobayashi-Ishihara, Hitoshi Takahashi, Kazuo Ohnishi, Kengo Nishimura, Kazutaka Terahara, Manabu Ato, Shigeyuki Itamura, Tsutomu Kageyama, Yasuko Tsunetsugu-Yokota

**Affiliations:** 1 Department of Immunology, National Institute of Infectious Diseases, Shinjuku, Tokyo, Japan; 2 Influenza Virus Research Center, National Institute of Infectious Diseases, Musashimurayama, Tokyo, Japan; 3 Tsuruga Institute of Biotechnology, Toyobo, Co., Ltd., Tsuruga, Fukui, Japan; 4 Department of Medical Technology, School of Human Sciences, Tokyo University of Technology, Ohta-ku, Tokyo, Japan; University of Georgia, United States of America

## Abstract

There is an urgent need for a rapid diagnostic system to detect the H5 subtype of the influenza A virus. We previously developed monoclonal antibodies (mAbs) against the H5 hemagglutinin (HA) for use in a rapid diagnostic kit. In this study, we determined the epitopes of the anti-H5 HA murine mAbs OM-b, AY-2C2, and YH-1A1. Binding assays of the mAbs to different strains of H5 HAs indicated that OM-b and AY-2C2 cross-reacted with HAs from clades 1, 2.1.3.2, 2.2, and 2.3.4, whereas YH-1A1 failed to bind to those of clades 2.1.3.2 and 2.3.4. HA chimeras revealed that the epitopes for each of the mAbs were in the HA1 region. Analysis of escape mutants revealed that OM-b and AY-2C2 mAbs interacted mainly with amino acid residues D43 and G46, and the YH-1A1 mAb interacted with G139 and K or R140 of H5 HA. Multiple alignments of H5 HA protein sequences showed that D43 and G46 were very conserved among H5N1 HAs, except those in clade 2.2.1 and clade 7 (88.7%). The epitope for YH-1A1 mAb was highly variable in the HAs of H5N1, although it was well conserved in those of H5N2-N9. The OM-b and AY-2C2 mAbs could bind to the HAs of clades 1.1 and 2.3.2.1 that are currently epidemic in Asia, and we conclude that these would be effective for the detection of H5N1 infections in this region.

## Introduction

The H5N1 influenza virus is a global threat to birds and humans, and by January 2014, there had been 650 cases of infections in people, with 386 deaths [1]. The disease in humans is epidemic in Asian and African countries such as Vietnam, Indonesia, Cambodia, and Egypt. Infections by H5N1 in people are limited to those who had close contact with infected animals, although the range and severity of symptoms in humans is not clear. For example, meta-analysis of serological studies on human H5N1 infections indicates a large number of missed infections [2], [3]. Several reports have highlighted outbreaks of human-adapted H5N1 viruses, although the level of risk has not been fully ascertained [4]–[8].

Rapid diagnosis of H5N1 infections is essential because patients treated in the early stages of the disease have a significantly lower level of mortality [9], [10]. Human H5N1 infections are mostly diagnosed by RT-PCR, which requires a few hours and some expertise to obtain results. Rapid and simple systems for the immunological detection of viral antigens have also been developed; however, these kits can have a low sensitivity [11] and cross-reactivity with other subtypes [12], [13]. The development of a rapid and reliable detection system for H5N1 without the need for RNA extraction would help to deliver an earlier clinical diagnosis in more localized areas.

For these reasons, several monoclonal antibodies (mAbs) that specifically recognize hemagglutinins (HAs) from the H5 subtype influenza viruses (H5 HA) were previously created in the development of a rapid detection system for H5N1 [14]. However, the range of cross-reactivity to H5 HAs is unclear because H5N1 viruses are still evolving and diversifying into multiple lineages, which are classified into clades (0–9) and subclades on the basis of their HA genealogy [15]. It is important to understand the epitope and cross-reactivity of anti-H5 HA mAbs in the development of a broadly reactive H5N1 influenza diagnostic kit.

In this study, we determined the epitopes of anti-H5 HA mAbs, and evaluated their range of reactivity to different clades of human H5N1 viruses. This was achieved by assessing the cross-clade reactivity of wild-type HAs, assessing the recognition sites of HA chimeras by flow cytometry, and analyzing escape mutants.

## Materials and Methods

### Viruses and Cells

A/Vietnam/1194/2004 (clade 1), A/Vietnam/1203/2005 (clade 1), A/Indonesia/05/2005 (clade 2.1.3.2), A/Turkey/12/2006 (clade 2.2), and A/Anhui/01/2005 (clade 2.3.4) were provided by the National Institute of Biological Standards and Controls (NIBSC, UK). A/Vietnam/VP-12-03/2012 (clade 1.1) and A/Narita/1/2009 (H1N1) were isolated and provided by the National Influenza Center, Pasteur Institute, Vietnam, and the Influenza Virus Research Center (IRC), NIID, Japan, respectively. A/whooper swan/Hokkaido/4/2011 (clade 2.3.2.1) was provided by Hokkaido University [16]. Culturing of the infectious H5N1 virus was done in a biosafety level 3 (BSL3) facility at the IRC, NIID, Japan. Batches of 293T cells and Madin–Darby canine kidney (MDCK) cells were cultured in Dulbecco’s Modified Eagle’s Medium and Minimum Essential Medium (Invitrogen, Carlsbad, CA, US), respectively, supplemented with 10% fetal bovine serum and incubated in a 5% CO_2_ atmosphere at 37°C.

### Antibodies

The monoclonal antibodies (mAbs) OM-b, AY-2C2, and YH-1A1 were produced previously [14]. C179 mAb (TaKaRa, Japan) was used as a positive control [17], and mouse IgG1 (BD Biosciences, San Diego, CA) and IgG2a (mAb Nk1.1) [18], [19] were used as isotype controls for flow cytometry analyses.

### Expression Vectors

Total RNA was extracted from virus stocks, and the HA genes were amplified by RT-PCR using the following primers: RT primer (Uni12), 5′-AGCAAAAGCAGG-3′; PCR primer set: 5′-GTCGACATGGAGAAAATAGTGCTTCTTTTTGCA-3′ and 5′-GTCGACATGGAGAAAATAGTGCTTC-3′. The HA gene fragments were cloned into pIRES-hrGFP-1α (Agilent) or a modified pENTR11 vector (Invitrogen, Carlsbad, CA) that contains the *Tight* promoter from pRetroX-Tight Pur (Clontech, USA) upstream of the multi-cloning site (MCS) and IRES-hrGFP sequences from pIRES-hrGFP-1α downstream of the MCS. The cloned pENTR11 was then recombined into the pCSII-RfA-Ed vector using the Gateway system (Invitrogen). pCSII-RfA-Ed was generated by replacing the EGFP gene of pCSII-RfA-EG (provided by Dr. Miyoshi, RIKEN, Japan) with the DsRed-express gene as follows: KpnI (blunted)/NotI fragment of pDsRed-Express vector (BD Biosciences) was subcloned into XhoI (blunted)/NotI site of pCSII-EF-MCS (provided by Dr. Miyoshi). Then, ApaI fragment of the pCSII-EF-MCS containing the DsRed-express gene was inserted into ApaI sites of pCSII-RfA-EG.

pIRES-hrGFP-1α vectors harboring chimeric or escaped mutant HAs were generated by site-directed mutagenesis (KOD Plus Mutagenesis Kit, Toyobo, Japan) [20]. For domain swapping, the globular or HA2 regions were PCR amplified from the pIRES-hrGFP-1αvector harboring A/Vietnam/1194/2004 HA or A/Narita/1/2009 HA (mega-primers). Then the PCR product was used as a mega-primer to produce the chimeric HA vector according to the mega-priming method of the site-directed mutagenesis [21]. pIRES-hrGFP-1α vectors harboring the chimeric HA gene were subcloned into pCSII-RfA-Ed as described above. The pRetroX-Tet-Off Advanced vector (Clontech) was used to express a tetracycline-controlled transactivator to activate the Tight promoter-transcriptions [22].

### Overexpression of HA Proteins and Flow Cytometry

HA-expressing vectors were transfected or co-transfected with pRetroX-Tet-Off Advanced into 293T cells using Lipofectamine 2000. Cells were collected 24 h post-transfection (hpt) and mixed with anti-H5 mAbs (30 µg/mL) or C179 mAb (5 µg/mL). Cells were mixed with Alexa Flour 647-conjugated anti-mouse immunoglobulin (Jackson ImmunoResearch, West Grove, PA) and incubated on ice for 10 min, and then analyzed using a FACS Calibur flow cytometer (BD Bioscience).

### Western Blotting

Cells expressing HAs were lysed with RIPA buffer (10 mM Tris-HCl (pH 7.6), 1% Triton X-100, 1% sodium-deoxycholate, 0.1% SDS, 150 mM NaCl, 5 mM EDTA (pH 8.0)) supplemented with a protease inhibitor cocktail (Roche, Switzerland). Lysates were separated by SDS-PAGE under reducing conditions, and then transferred to a PVDF membrane (Amersham Biosciences, Piscataway, NJ). Membranes were blocked with Tris-buffered saline containing 0.05% Tween-20 and 0.3% skimmed milk, and then reacted for 1 hour with a mixture of sheep anti-H5 and H1 HA sera (anti-VN1194 HA and anti-A/New Caledonia/20/99 (IVR-116) HA sera, provided by IRC, NIID, Japan). The membrane was then incubated for 30 min with HRP-conjugated anti-goat/sheep immunoglobulin or anti-mouse immunoglobulin antibodies (Jackson ImmunoResearch).

### Selection of Escape Mutant Viruses

The A/Vietnam/1194/2004 vaccine strain virus (10^6^ TCID_50_/50 µL) was separately incubated with each mAb (OM-b, 0.625 µg/mL; AY-2C2, 0.313 µg/mL; YH-1A1, 0.078 µg/mL) for 30 min at 37°C. Mixtures were serially diluted and used to infect MDCK cell layers in a 96-well microtiter plate. At 5–7 days post-infection, the supernatants of cells with cytopathic effects were harvested. The procedure was repeated twice and the final supernatants were then added to MDCK cells in a 6-well plate. Escape mutants were isolated using a plaque assay as described previously [23]. Total RNA was extracted from the escape mutants and used as the template for RT-PCR amplification of the HA gene using the primers described in the Expression vectors subsection. The nucleotide sequences of the HA gene PCR products were analyzed by direct sequencing.

### Neutralization Titers of mAbs with Escape Mutant Viruses

A confluent monolayer of MDCK cells was prepared in each well of a 96-well microtiter plate. Each of the mAbs were mixed with 100 TCID_50_ of the escape mutant virus in the presence of trypsin and incubated at 37°C for 30 min [24]. Suspensions of the viruses were individually added to the MDCK cells and incubated for 7 days at 34°C in 5% CO_2_. Cells were then fixed with 10% formaldehyde, and stained with NB solution (0.1% naphthol blue black, 0.1% sodium acetate, and 9% acetic acid) before measuring OD_630_. Cell viability was calculated using a calibration of the OD_630_ values from uninfected and virus-only wells as 100% and 0%, respectively. The concentration of the final dilution that reduced the cytopathic effects of the virus by 50% was taken as the neutralizing titer.

### *In silico* Analysis of HA Structures

The crystal structures of VN1194 HA (PDB ID: 2IBX) and H1pdm HA (PDB ID: 3LZG) were analyzed by *in silico* modeling, and root-mean-square deviation (RMSD) measurements [25] were determined using *Molecular Operating Environment (MOE)* ver. 2013.08 software (Chemical Computing Group Inc., Canada). To model chimeric HAs, each domain of H5 HA was grafted onto that of H1pdm HA with a minimal energy conformation.

### Statistics

Experiments were independently conducted three times. The data was analyzed using a Student’s t-test and p-values of <0.05 were considered statistically significant. Error bars represent standard deviations.

## Results

### Cross-clade Reactivities of H5N1 HAs

Anti-H5 HA mAbs were produced by immunizing BALB/c mice with inactivated VN1194 viruses [14]. The characteristics of OM-b, AY-2C2, and YH-1A1 mAbs used in this study are shown in Table 1. Each mAb had micro-neutralization (MN) activity but no hemagglutination inhibition (HI). It was previously proposed that the AY-2C2 mAb recognizes a conformational epitope [14].

**Table 1:** Characteristics of anti-HA mAbs.

mAb	JH gene	Subclass*	HI activity	MN activity (µg/50 µL)*	Clade dependence*
OM-b	4	G2a	-	0.625	No
AY-2C2	2	G1	-*	0.313	No
YH-1A1	4	G2a	-*	0.078	Yes

*Results reported previously by Ohnishi *et al.* [14].

**Figure 1:**
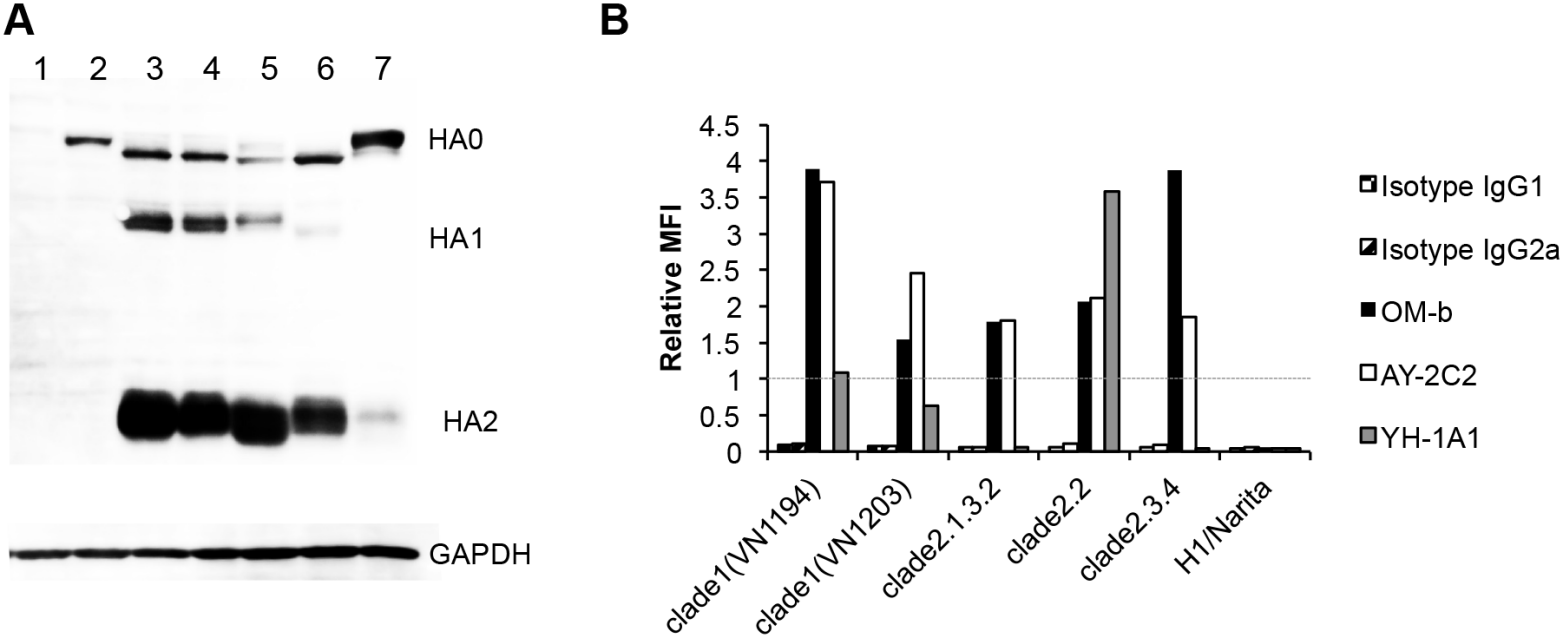
Cross-clade binding activities of the four mAbs. (A) Expression patterns of HA proteins. H5 HA expression vectors were transfected into 293T cells. Cells were collected at 24 hpt and analyzed by Western blotting. Sheep anti-H5 HA and H1 HA polyclonal sera were used to detect HAs (upper panel). Levels of glyceraldehyde-3-phosphate dehydrogenase (GAPDH) expression are shown as a loading control (lower panel). Cells transfected with an empty vector (lane 1) and the expression vectors of H1pdm HA (lane 2), VN1194 HA (lane 3, clade 1), VN1203 HA (lane 4, clade 1), clade 2.1.3.2 HA (lane 5), clade 2.2 HA (lane 6), and clade 2.3.4 HA (lane 7) were analyzed. (B) Interactions between the H5 HAs and mAbs. The cells described in (A) were incubated with mAbs and the reactivity was determined by flow cytometry. The MFI of each mAb was normalized against that of mAb C179 (1.0) to account for different expression levels. A representative result of three independent experiments with high reproducibility is shown.

The binding of anti-H5 HA mAbs to different clades of H5 HAs was determined using a flow cytometer to understand the breadth of cross-reactivity. The HA genes from five representative H5N1 strains were cloned into expression vectors and transfected into 293T cells. Western blot analysis of HA-expressing cells using a mixture of polyclonal anti-H5 and anti-H1 HA sera revealed that precursor HA0 was present in all transfectants. Cleaved HA1 and HA2 proteins were detected in H5 HA transfectants, although clade 2.3.4 HA showed weak cleavage activity (Figure 1A), and is consistent with the findings of Tang *et al*. [26].

These transfectants were reacted with the anti-H5 HA (OM-b, AY-2C2, and YH-1A1) mAbs and analyzed by flow cytometry. The ratio of the mean fluorescence intensity (MFI) of the mAb relative to that of the C179 mAb, which broadly recognizes HAs of group 1 influenza viruses, was determined to compare the binding affinity of each transfectant (Figure 1B). Isotype mAbs were used as controls for background staining. YH-1A1 mAb bound to clade 1 and 2.2 HAs, but not to clade 2.1.3.2 or 2.3.4 HAs. OM-b and AY-2C2 mAbs bound to HAs from all clades. YH-1A1 mAb bound strongly to clade 2.2 HA, but weakly to the clade 1 (VN1194) HA that is used for immunization. Of the mAbs, OM-b bound the strongest to clade 2.3.4 HA. The results suggest that OM-b and AY-2C2 mAbs recognize the well-conserved regions of H5 HA, whereas YH-1A1 mAb interacts with variable regions.

### Mapping of the H5 HA Domain Recognized by anti-H5 HA mAbs

**Figure 2:**
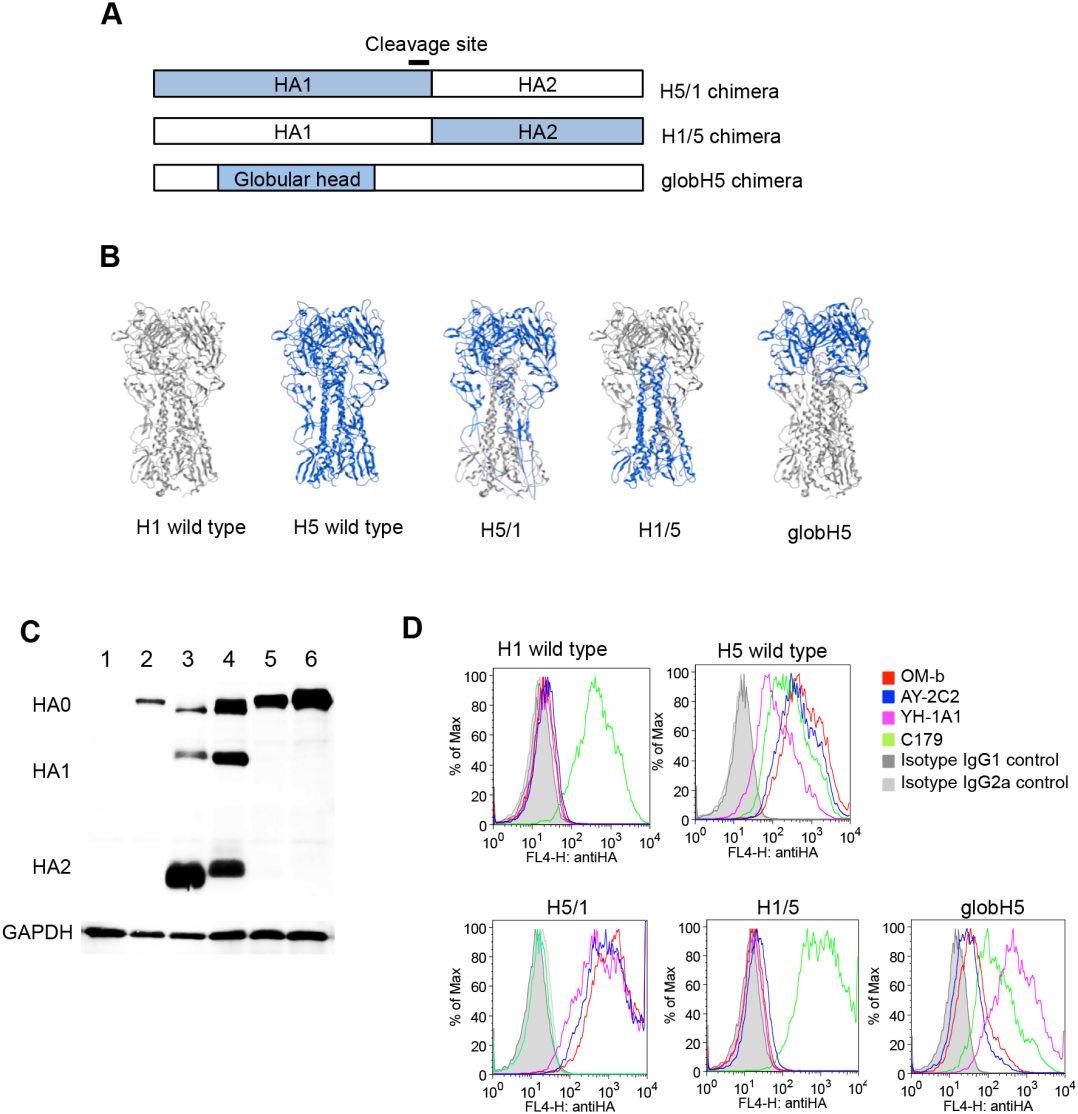
Epitope screening with HA chimeras. (A) Schematic diagram of chimeric HAs. A series of chimeric HAs were generated by domain swapping between VN1194 HA (blue) and H1pdm HA. (B) Formations of chimeric HAs simulated by MOE based on the crystal structure of VN1194 HA (PDB ID: 2IBX, blue) and H1pdm HA (PDB ID: 3LZG, gray). (C) Expression of the chimeric HAs in mammalian cells. The chimeric HA expression vectors were co-transfected with pRetroX-Tet-Off Advanced into 293T cells. Cells were collected at 24 hpt and analyzed by Western blotting. Upper panel, expression patterns of the HAs; lower panel, loading control (GAPDH). Cells transfected with an empty vector (lane 1) and the expression vectors of H1pdm HA (lane 2), VN1194 HA (lane 3), H5/1 chimera (lane 4), H1/5 chimera (lane 5), and globH5 chimera (lane 6) were analyzed. (D) Interaction between the chimeric HAs and OM-b, AY-2C2 and YH-1A1mAbs. Cells described in (B) were incubated with mAbs, and the reactivity was analyzed by flow cytometry. Histograms of levels of reactivity against each mAbs in cells expressing the indicating HAs.

Three plasmids expressing chimeric HAs were constructed by domain swapping between the VN1194 HA and H1pdm HA (Figure 2A) to identify the precise binding regions of the mAbs. The H5/1 chimera has amino acid sequences corresponding to that of VN1194 HA in the HA1 region and that of H1pdm HA in the HA2 region. The H1/5 chimera has H1pdm HA1 and VN1194 HA2 regions. The globH5 chimera has a VN1194 globular head region and other regions derived from H1pdm HA (amino acids 42–275 of H1pdm HA replaced with amino acids 42–274 of VN1194 HA, GenBank: ABP51976 and ACR09396, respectively). Computer simulation revealed that the conformation of the HA1 stem loop region of the H5/1 chimera was different to that of wild-type H5 HA (Figure 2B and Figure S1).

The RMSD of Cα indicated that the globular head region of the chimera was similar to that of wild-type H5 HA (RMSD = 1.606 angstrom (Å)). The H1/5 chimera maintained the conformation of the stem and globular head regions corresponding to the original H5 and H1 subtypes, respectively (RMSD = 0.488 Å). The globH5 chimera also appeared to maintain conformation of the original H5 and H1 HAs, except around the boundary region between the globular head and stem (RMSD of the boundary region = 2.866 Å, and RMSD of the other region = 1.672 Å).

Plasmids of the chimeric HAs were transfected into 293T cells, and their expression was analyzed by Western blotting using a mixture of polyclonal goat sera against H5 and H1 HA (Figure 2C). Consistent with the wild-type VN1194, H5/1 maintained cleavage activity without trypsin treatment. This was because the cleavage site was derived from the VN1194 HA (Figure 2A). The binding of mAbs to each HA chimera was assessed by flow cytometry (Figure 2D), and the results are summarized in Table 2. C179 mAb bound to the globH5 and H1/5 chimeras, but not to H5/1. OM-b, AY-2C2, and YH-1A1 mAbs interacted well with the H5/1 chimera, but were unable to bind to H1/5, confirming their recognition of the HA1 domain. These three mAbs also bound to the globH5 chimera; the strength of attachment was greater for YH-1A1 than for OM-b and AY-2C2. Thus, the results indicate that the mAbs recognize the globular region of the H5 HA with different affinities.

**Table 2:** Summary of the binding of mAbs to chimeric HAs.

HA	OM-b	AY-2C2	YH-1A1	C179
H5 HA	++	++	+	++
H1 HA	−	−	−	++
H5/1 chimera	++	++	++	−
H1/5 chimera	−	−	−	++
globH5 chimera	+	+	++	++

Differences in the MFIs to HAs between the mAbs and its isotype control were categorized as follows: over 300 (++), between 20 and 300 (+), and under 20 (–).

### Mab Binding Sites

Escape mutant viruses were selected after a second round of infection of VN1194 vaccine strains in the presence of each mAb to deduce which amino acid positions may contribute to the interaction with H5 HA. Results of nucleotide sequencing of the HA coding region are shown in Table 3. At amino acid position 43 in the escape mutants against AY-2C2 mAb, substitutions of D to N and D to Y were found at a similar frequency. The same amino acid substitutions at D43N and D43Y were present in the escape mutants against OM-b mAb. D45Y and G46E substitutions were also observed in escape mutants of OM-b mAb. The escape mutants against YH-1A1 mAb harbored G139E or K140E substitutions.

**Table 3:** Mutation sites in the HA gene of virus escape mutants against each mAb.

mAb	Escape mutation		Frequency (%)
	Amino acid	Nucleotide	
OM-b	D43N	G127A	83.32
	D43Y	G127T	5.56
	D45Y	G133T	5.56
	G46E	G137A	5.56
AY-2C2	D43N	G127A	57.14
	D43Y	G127T	42.86
YH-1A1	G139E	G416A	60.00
	K140E	A418G	40.00

The mutations occurred singly in each escape mutant.

The MN activity of each mAb to the escape mutants was determined (Table 4). The escape mutants of OM-b and AY-2C2 were resistant to neutralization by OM-b and AY-2C2 mAbs, but not by YH-1A1 mAb. However, resistance of the AY-2C2 escape mutants against the OM-b and AY-2C2 mAbs was weak. By contrast, the escape mutants of YH-1A1 mAb became resistant to neutralization only by YH-1A1 mAb. The results shown in Tables 3 and 4 indicate that OM-b and AY-2C2 have major epitopes at D43, D45, and G46. YH-1A1 has major epitopes at G139 and K140.

**Table 4:** Neutralization titers of mAbs with escape mutants of the VN1194 virus.

Escape mutant		MN activity*		
mAb	Mutation	OM-b	AY-2C2	YH-1A1
OM-b	D43N	−	−	+
	D43Y	−	−	±
	D45Y	−	−	+
	G46E	−	−	+
AY-2C2	D43N	±	±	+
	D43Y	±	±	+
YH-1A1	G139E	+	+	−
	K140E	+	+	−

*Differences between the micro-neutralization (MN) titers of mAbs in reactions with the wild-type VN1194 virus and its escape mutants. +, MN titer of the mAb does not differ from that with the wild-type virus; ±, MN titer is 2-8-fold greater than that with the wild-type virus; −, MN titer is at least 16-fold greater than that with the wild-type virus.

**Figure 3:**
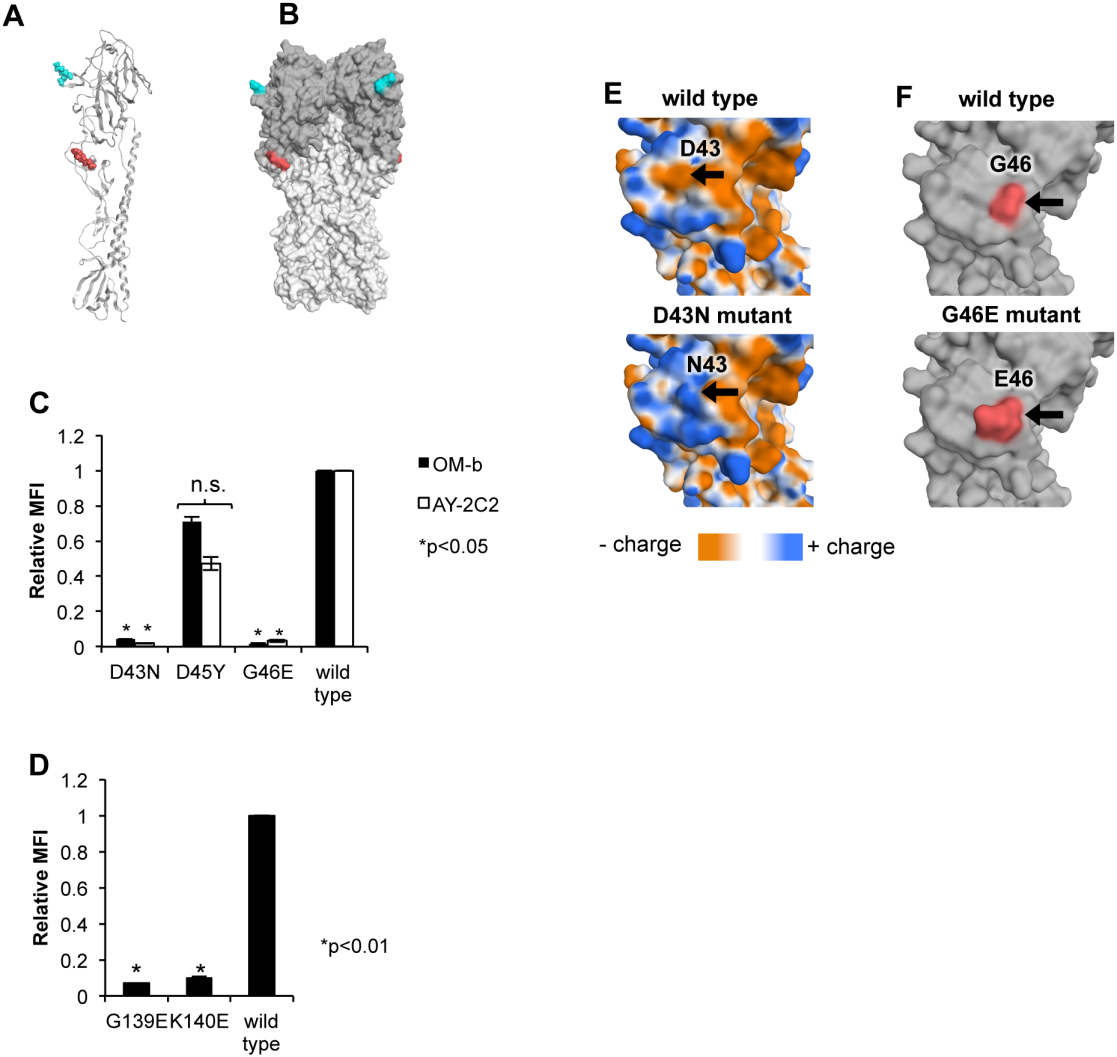
Binding of the mAbs to escape mutant HAs. (A and B) Structures of the mutation sites. Amino acid mutation sites are mapped onto the ribbon structure of the H5 HA monomer (A) and a surface representation of trimeric H5 HA (B). The globular region is colored dark gray. Sites of escape mutations against AY-2C2 and OM-b mAbs are colored red, and those against YH-1A1 mAb are colored blue. (C and D) Results of flow cytometry. A series of pIRES-hrGFP-1α vectors expressing the mutant HA were transfected into 293T cells. At 24 hpt, the cells were collected and incubated with the OM-b (C), AY-2C2 (C), or YH-1A1 (D) mAbs, and the binding activities were determined by flow cytometry. The MFI of each mAb was normalized against that of the C179 mAb to account for different expression levels. The relative MFI of each mAb to that of the wild-type HA is shown. P-values were calculated by Student’s t-test. No significant differences with the wild type is denoted by n.s. Standard deviation is represented by vertical bars. (E) Surface of the D43N mutant. Surface maps of wild-type VN1194 HA (upper panel) and D43N mutant HA (lower panel) are shown. The mutant formation was simulated by MOE based on the crystal structure of the VN1194 HA monomer (orange, negatively charged area; white, neutral area; blue, positively charged area). (F) Surface of the G46E mutant. Surface maps of wild-type VN1194 HA (upper panel) and G46E mutant HA (lower panel) are shown. (G46 and E46 are shown in red).

The three-dimensional structures of the mutation sites of H5 HA were analyzed using the MOE program. Epitope mapping of H5 HA (PDB ID: 2IBX) revealed mutations at the origin of the globular head (Figure 3A and B, red). The YH-1A1 mutations were located on the surface of the globular domain a short distance from the receptor-binding site (Figure 3A and B, blue).

The escape mutant HAs (D43N, D45Y, G46E, G139E, and K140E) were overexpressed in 293T cells and analyzed by flow cytometry (Figure 3C and 3D) to determine whether conformational changes affect the binding sites of mAbs. Isotype IgG1 or IgG2a was used as a background control (data not shown). OM-b and AY-2C2 mAbs could not bind with the G46E and D43N mutants (Figure 3C). However, there was no significant change in the binding activities between the D45Y mutant and OM-b or AY-2C2 mAbs. YH-1A1 mAb did not interact with the G139E or K140E mutant HAs (Figure 3D).

The computer simulation revealed that binding of the different mAbs was affected by altering the physicochemical status of the H5 HA molecule. A shift in the charge from negative to positive at the D43N mutation site occurred without altering the size of the side chains (Figure 3E) [27]. The G46E and G139E mutations appear to change the surface structure at this particular site (Figure 3F for G46E and data not shown for G139E). The K140E mutation appeared to change the charge from positive to negative, and also affected the shape of the surface (data not shown).

The results of this study revealed that the binding sites of OM-b and AY-2C2 mAbs overlapped and were located at D43 and G46, whereas those of YH-1A1 mAb were located at G139 and K140.

### Epitope Conservation among H5 Influenza Viruses

Epitope sequences of the escape mutants were compared with H5N1 strains (Table 5). The D43, D45, and G46 epitopes of OM-b and AY-2C2 mAbs, and the G139 epitope of YH-1A1 mAb, were conserved among all the clades. The K140 epitope of YH-1A1 was variable. YH-1A1 mAb interacted with clade 1 and clade 2.2 (K140 and R140, respectively), but not with clade 2.1.3.2 and 2.3.4 HAs, as shown in Figure 1B. YH-1A1 may preferentially bind to a positively charged amino acid at the 140^th^ position, but not to one that is neutrally or negatively charged, such as serine, threonine, and glutamine acid.

**Table 5:** Alignments of the epitope sequences among H5N1 strains.

Interacting antibody		OM-b/AY-2C2 epitope*				YH-1A1 epitope*	
Amino acid sequence (H5 numbering)		43	45	46	139		140
	A/Vietnam/1194/2004 (1)	D	D	G	G		K
	A/Vietnam/1203/2004 (1)	D	D	G	G		K
Human	A/Indonesia/05/2005 (2.1.3.2)	D	D	G	G		*S*
strain	A/Turkey/12/2006 (2.2)	D	D	G	G		R
(clade)	A/Anhui/01/2005 (2.3.4)	D	D	G	G		*T*
	A/Vietnam/VP-12-03/2012 (1.1)	D	D	G	G		*Q*
	Escape mutants	*N*	Y	*E*	*E*		*E*
Avian strain (clade)	A/whooper swan/Hokkaido/4/2011 (2.3.2.1)	D	N	G	G		*N*
**Conservation rate among human and avian H5N1 (%)**		**88.8**	**87.4**	**99.9**	**99.2**		**60.8**

*Amino acids in italic letters are critical changes for OM-b/AY-2C2 or YH-1A1 binding.

**Figure 4:**
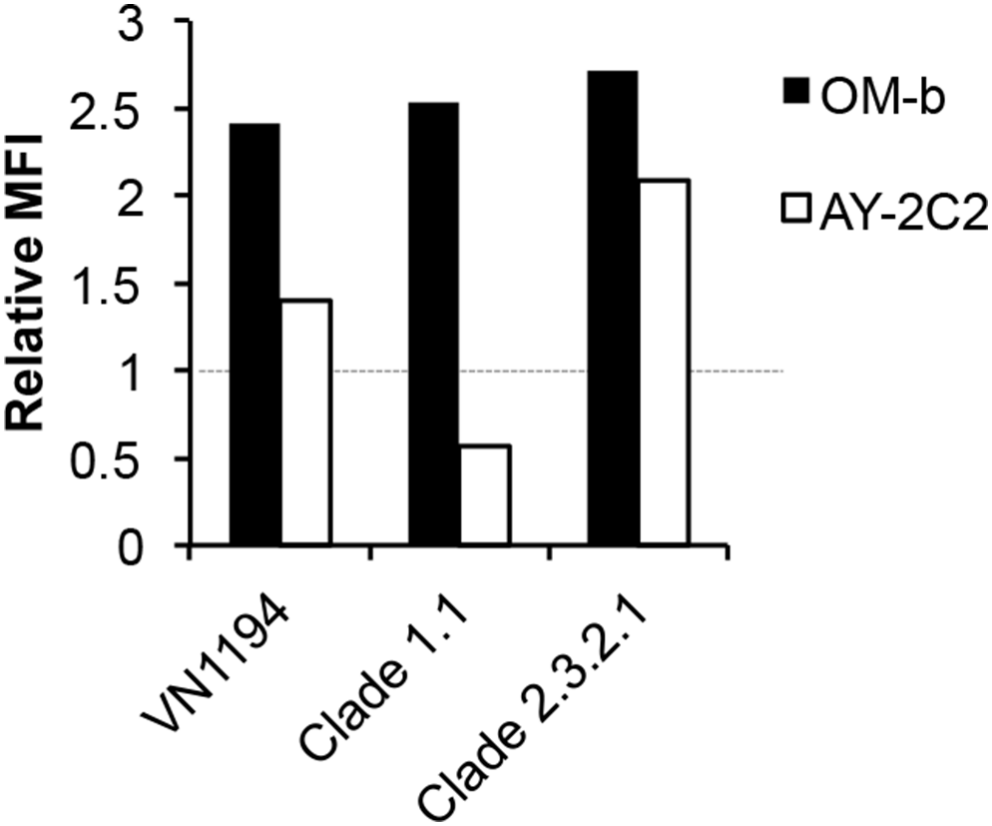
Binding of HAs from the recent H5N1 epidemic. Clades 1.1 and 2.3.2.1 HA genes were subcloned into pIRES-hrGFP-1α. HAs were overexpressed in 293T cells, and the binding of the mAbs was determined by flow cytometry. The MFI of each mAb was normalized against that of the C179 mAb (1.0) to account for different expression levels. A representative result of three independent experiments with high reproducibility is shown.

The ability of OM-b and AY-2C2 mAbs to recognize H5N1 strains that are currently epidemic in humans and birds (i.e., clades 1.1 and 2.3.2.1) was determined. A/Vietnam/VP-12-03/2012 (clade 1.1) maintains the OM-b and AY-2C2 epitope sequences, and A/whooper swan/Hokkaido/4/2011 (clade 2.3.2.1) harbors a D45N substitution. These HAs were expressed in 293T cells, and attachment of the OM-b and AY-2C2 mAbs to the HAs was determined by flow cytometry. OM-b and AY-2C2 mAbs could bind to the clade 1.1 and 2.3.2.1 HAs (Figure 4). The results indicate that D43 and G46, but not D45, are important for recognition by OM-b and AY-2C2 mAb, and that these mAbs could be used to detect current H5N1.

Alignments of influenza HA amino acid sequences from the Influenza Virus Resource database [28] showed that the conservation rates of the epitope sites among human and avian H5N1 strains were 88.8% (D43), 99.9% (G46), 99.2% (G139), and 61.0% (K140 or R140) (Table 5). The overall epitope conservation rates for H5 influenza viruses in the database are estimated in Table 6. The conservation rate of human and avian H5N1 in the OM-b and AY-2C2 epitope sequences (D43 and G46) was 88.7% and 60.6% for YH-1A1 (G139 and K or R140). By contrast, the conservation rate of OM-b and AY-2C2 epitope sequences among avian H5N2-N9 strains was as low as 0.74%. This may be because the amino acid sequences of H5N2-N9 HAs, except H5N5, contain serine at the 43^rd^ position, which appears to change the electrostatic potential in a manner similar to the D43N mutation. Alternatively, the YH-1A1 epitope sequences are highly conserved (94.0%), with most of the H5N2-N9 HA sequences containing glycine at the 139^th^ position and arginine at the 140^th^ position. These results suggest that OM-b and AY-2C2 mAbs can specifically detect H5N1 infections.

**Table 6:** Conservation of the epitopes among H5 Influenza viruses.

HA subtype	Virus host	OM-b and 2C2 epitope sequences/total number	Conservation rate (%)	
H5N1	Human	277/324	85.5	88.7
H5N1	Avian	2362/2652	89.1	
H5N2-N9	Avian	6/808	0.74	
H1N1pdm	Human, avian	0/1224	0	
**HA subtype**	**Virus host**	**1A1 epitope sequences/total number**	**Conservation rate (%)**	
H5N1	Human	212/327	64.8	60.6
H5N1	Avian	1615/2701	59.8	
H5N2-N9	Avian	725/769	94.3	
H1N1pdm	Human, avian	0/1224	0	

The results also revealed that the OM-b and AY-2C2 epitope was conserved among 85.5% of human H5N1 strains (Table 6). The remaining 14.5% of H5N1s correspond to one strain in clade 2.1.3.2 that harbors a G46R substitution (A/Indonesia/CDC582/2006), and the majority of clade 2.2.1 that are currently epidemic in Egypt and contain D43N substitutions [29], [30]. We consider that the OM-b and AY-2C2 mAbs will cover human H5N1 infections emergent in Asia, but not those in Africa. Similarly, the results of the epitope conservation analysis in avian H5N1 indicate that the mAbs cannot cover sublineage B viruses of clade 2.2.1, and clade 7, 7.1, and 7.2 viruses, which also harbor D43N substitutions.

## Discussion

There is an urgent need to develop a diagnostic system that is highly sensitive and rapid for the detection of the H5 subtype influenza virus. Several systems that can rapidly detect this virus were recently developed [31], [32]; however, the range of effectiveness for these approaches is unknown. We recently developed a chemiluminescent ELISA-based rapid H5N1 influenza diagnostic system using OM-b mAb and demonstrated its effectiveness at screening for the infection prior to analysis of the virus RNA at a centralized laboratory (unpublished data). In this study, we determined the epitopes of our anti-H5 HA mAbs, including OM-b, and characterized their breadth of cross-reactivity. Our findings suggest that the highly conserved nature of the OM-b epitope make it ideal to detect Asian strains of the virus.

Analysis of chimeras revealed that the H5 HA-specific mAbs interact with the HA1 of H5 HA. Escape mutant analysis showed that OM-b and AY-2C2 mAbs interacted with the origin of the globular region, D43 and G46, corresponding to the antigenicity-associated site C, while the YH-1A1 mAb reacted with the protrusion from the globular head surface, G139 and K140, corresponding to the antigenicity-associated site A [33], [34]. Antigenic drift events of H5N1 favor the residues in antigenic sites A, B, and D rather than those in C and E [33]. Consistent with this, our results also showed that OM-b and AY-2C2 mAbs are likely to recognize more strains of H5N1 (88.7% coverage) than YH-1A1 mAb (60.6% coverage).

The results from our previous and present studies indicate that OM-b, AY-2C2 and YH-1A1 mAbs bind to the globular region, and that they have MN, but not HI, activities [14]. Consistent with this, there are several anti-HA mAbs that attach to the globular head other than the receptor-binding site and can neutralize virus infectivity without HI activity [35]. Their mode of action is thought to involve inhibiting the post-acidification conformational change of HA proteins, which is essential for virus entry [27]. The mechanism by which OM-b, AY-2C2 and YH-1A1 mAbs neutralize infection by the virus remains unclear and warrants further investigation.

The binding strengths of OM-b and AY-2C2 mAbs to the globH5 chimera were weaker than those of the other mAbs. This may be because the epitope is located close to the site of exchange. Computer simulation of the structure indicated that the ruggedness and charge of the HA surface, particularly around the epitope sites, differ between globH5 and wild-type H5 HA (data not shown). The widely cross-reactive C179 mAb did not bind to the H5/1 chimera, despite the presence of the C179 mAb epitope [17], [36]. This may have a structural explanation because the folding pattern of the HA1 stem loop of the H5/1 chimera was notably different from that of the wild-type HA (Figure 2B and Figure S1). An increased distance of the main epitope sites of the C179 mAb could result in poorer binding to the H5/1 chimera (distances between T318 of HA1 and V52 of HA2 are 22.74Å in H5/1 HA, and 11.04Å in the wild-type H5 HA).

D43 and G46 affect the affinities of OM-b and AY-2C2 mAbs, although the frequency of the escaped mutant virus at G46 was low or not isolated. G46 may be an important location that governs the structure and function of the HA, and accordingly, it may be difficult for a mutation to occur at that site. This may explain why the OM-b and AY-2C2 epitopes are highly conserved among human and avian H5N1 (99.9% identity).

Despite the inability of the OM-b and AY-2C2 mAbs to neutralize the D45Y virus, they were able to bind with the D45Y HA mutant and clade 2.3.2.1 HA containing a D45N substitution. Recent studies characterizing escape mutants against anti-HA antibodies reported examples where HAs promote compensatory mutations in neuraminidase (NA) [37], [38] or increase HA receptor-binding avidity [39] to acquire resistance against antibody-binding pressure. This may explain why the D45Y virus is resistant to OM-b and AY-2C2 mAbs. D45 is not likely to have a marked effect on the HA binding of these mAbs, but is likely to have an impact on the infectivity in the presence of OM-b or AY-2C2 mAbs. It is also possible that OM-b and AY-2C2 mAbs can bind with the D45Y HA on the cell surface, but not on the viral particle. In this instance, the NA protein may play a role in antibody binding; however, we have observed (ELISA and Western blot) that OM-b and AY-2C2 did not bind to a recombinant NA derived from the VN1194 virus [14].

There were differences in the MN activities of OM-b and AY-2C2 to the D43N/Y viruses selected by OM-b and those selected by AY-2C2. The HA titers of the D43N/Y viruses selected by AY-2C2 were low compared with those selected by OM-b at the same infectivity titers (data not shown). We consider that differences between the MN titers of escape mutants D43N/Y selected by OM-b and those selected by 2Y-2C2 may be due to changes in other genes such as NA [37], [38].

The mAbs used in this study were specific to H5 HAs, and did not recognize other subtype viruses [14], with OM-b and AY-2C2 having a broad cross-reactivity among the H5N1 HAs in clades 1, 2.1.3.2, 2.3.4 [14], 2.2, 1.1, and 2.3.2.1. However, the OM-b and AY-2C2 coverage of human and avian H5N1 strains isolated from Africa in 2010–2013 was only 20.2% (data not shown). This is probably because the mAbs did not recognize several of the evolved clade 2.2.1 viruses [30], [40]. Of note, we previously showed that OM-b and AY-2C2 mAbs were able to bind with A/turkey/Turkey/1/2005, which classified as an early clade 2.2.1 virus [14]. Also, OM-b mAb recognized additional 8 recombinant HA proteins originated from Asian H5N1 strains (clade 0, 1, 2.1.1, 2.2 and 2.3.4, all harboring 43D, 45D or N and 46G, unpublished data). Because the coverage of the Asian H5N1 strains was estimated to be as high as 96.5% (see Figure S2), we believe that OM-b and AY-2C2 mAbs are effective for the detection of H5N1 viruses in this geographical area. These observations are consistent with other studies of murine mAbs among these viruses [27], [40]. To develop the methodology further, mAbs that recognize H5 HAs with D43N substitutions are needed to widen the range of detection for H5N1 strains.

## Supporting Information

Figure S1 **Structural comparison between H5 HA and chimeric HA.** Loop structures of H5/1 chimeric HA (gray and blue, in the same order as Figure 3A) and VN1194 wild-type HA (orange) are superimposed. Left-hand diagram shows the structures of monomeric HAs. The main epitope sites of the C179 mAb are shown as blue (T318) and gray (V52) spheres. Orange spheres represent the wild-type HA. Right-hand diagram shows the structures of trimeric HAs.
(TIFF)

Figure S2 **OM-b and AY-2C2 epitope conservation in human and avian H5N1 strains isolated in Asia during 2010–2013.** (A) Distribution of the OM-b and AY-2C2 epitopes in Asia and the regional breakdown of non-conserved strains. HA sequences of the Asian H5N1 epidemic of 2010–2013 (Influenza Virus Resource database [27]) were multiple-aligned and analyzed for conservation of D43 and G46. (B) List of non-conserved strains.
(TIF)
