# Supplementary figures and images for: Broad Cross-Reactive Epitopes of the H5N1 Influenza Virus Identified by Murine Antibodies against the A/Vietnam/1194/2004 Hemagglutinin
Source: PLoS One. 2014 Jun 19;9(6):e99201. doi: 10.1371/journal.pone.0099201 (PMC4063728; doi:10.1371/journal.pone.0099201)

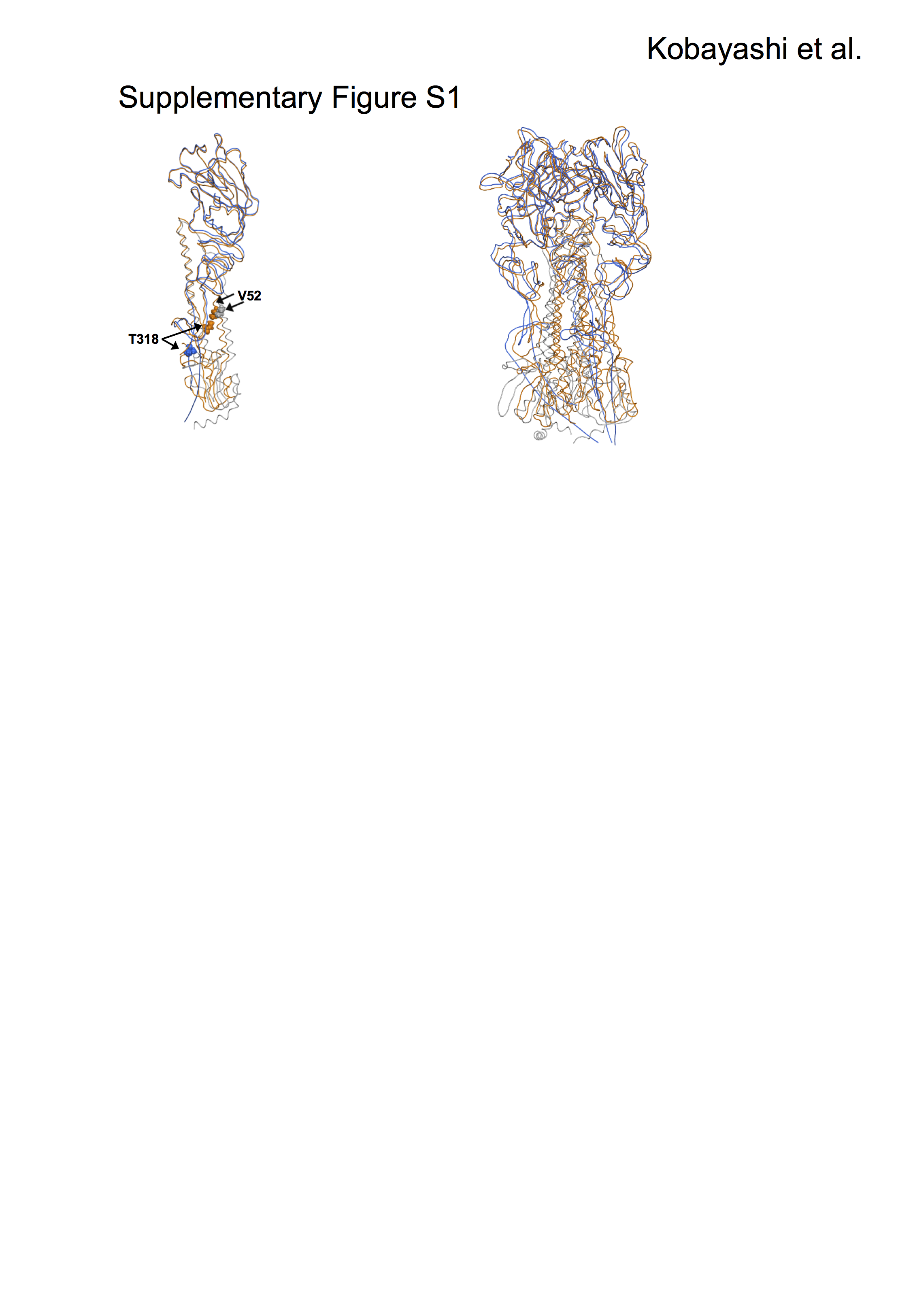

Supplement: Figure S1 — Structural comparison between H5 HA and chimeric HA. Loop structures of H5/1 chimeric HA (gray and blue, in the same order as Figure 3A) and VN1194 wild-type HA (orange) are superimposed. Left-hand diagram shows the structures of monomeric HAs. The main epitope sites of the C179 mAb are shown as blue (T318) and gray (V52) spheres. Orange spheres represent the wild-type HA. Right-hand diagram shows the structures of trimeric HAs. (TIFF) [file pone.0099201.s001.tif]

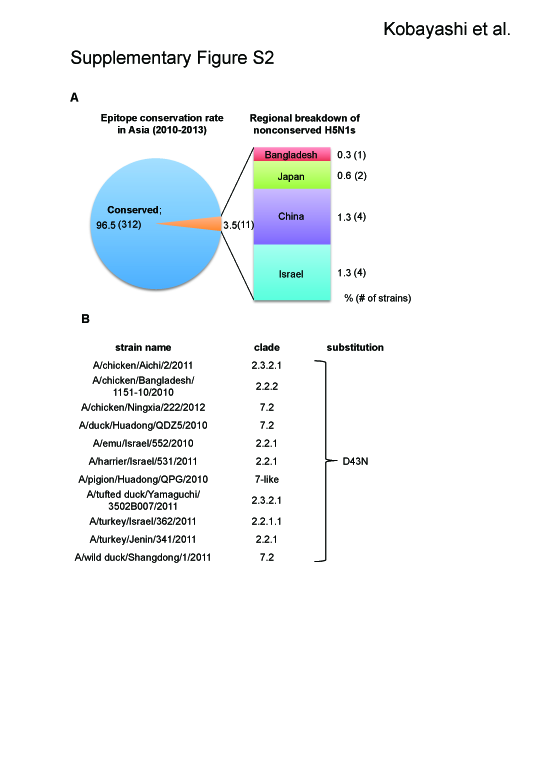

Supplement: Figure S2 — OM-b and AY-2C2 epitope conservation in human and avian H5N1 strains isolated in Asia during 2010–2013. (A) Distribution of the OM-b and AY-2C2 epitopes in Asia and the regional breakdown of non-conserved strains. HA sequences of the Asian H5N1 epidemic of 2010–2013 (Influenza Virus Resource database [27]) were multiple-aligned and analyzed for conservation of D43 and G46. (B) List of non-conserved strains. (TIF) [file pone.0099201.s002.tif]
